# Angiogenic Ability of Extracellular Vesicles Derived from Angio-miRNA-Modified Mesenchymal Stromal Cells

**DOI:** 10.1007/s13770-025-00741-w

**Published:** 2025-07-31

**Authors:** Yoshiki Wada, Toshifumi Kudo, Anri Koyanagi, Tomomi Kusakabe, Ayako Inoue, Yusuke Yoshioka, Takahiro Ochiya, Shoji Fukuda

**Affiliations:** 1 Department of Vascular Surgery, Institute of Science Tokyo, Tokyo, Japan; 2 Department of Comprehensive Pathology, Institute of Science Tokyo, Tokyo, Japan; 3 Center for Cell Therapy and Regenerative Medicine, Tokyo Medical University, 6-7-1, Nishishinjuku, Shinjuku-ku, Tokyo, 160-0023 Japan; 4 Department of Molecular and Cellular Medicine, Tokyo Medical University, Tokyo, Japan; 5 Department of Cardiovascular Surgery, Tokyo Medical University, Tokyo, Japan

**Keywords:** Angiogenesis, Extracellular vesicles, MicroRNAs, Chronic limb-threatening ischaemia

## Abstract

**Background:**

Regenerative therapy using extracellular vesicles (EVs) is a promising approach for the supportive treatment of chronic limb-threatening ischaemia. Herein, we examined the angiogenic potential of EVs derived from genetically modified mesenchymal stromal cells (MSCs), focusing on the angio-micro RNAs (miRNAs) in EVs.

**Methods:**

Bone marrow-derived MSCs (BM-MSCs) were transfected with lentiviral vectors containing specific angio-miRNAs (miRNA-126, -135b, or -210), and miRNA overexpression was confirmed using quantitative polymerase chain reaction (qPCR). EVs were isolated from the BM-MSC culture medium and characterised using fluorometry, nanoparticle tracking analysis, and ExoScreen assays. *In vitro,* human umbilical vein endothelial cells (HUVECs) were used to evaluate the angiogenic potential of the EVs. *In vivo*, EVs were injected into the ischaemic hindlimb muscles of mice, and limb ischaemia severity, blood perfusion, and histological analysis of muscle tissue were performed.

**Results:**

qPCR analysis confirmed the overexpression of angio-miRNAs in MSCs transfected with lentiviral vectors. Isolated EVs expressed CD63 and had consistent protein-to-particle ratios. Tube formation was significantly enhanced when HUVECs were cultured with EV126, EV135b, or their combination (EV126 + EV135b) (*p* < 0.05), compared to BM-MSC co-culture. *In vivo*, only the double and triple EV groups significantly improved limb perfusion compared to the EVcontrol (*p* < 0.05); single EVs showed no significant difference. Histological analysis showed increased capillary density in ischaemic muscles following injection of combined EVs.

**Conclusion:**

EVs derived from genetically modified MSCs promoted angiogenesis both *in vitro* and *in vivo*, with a combination of modified EVs demonstrating significantly superior therapeutic effects than single or native EVs.

## Introduction

Chronic limb-threatening ischaemia (CLTI) has a one-year lower limb amputation rate of 22% and a mortality rate of 22% when revascularisation is unavailable [1]. These outcomes negatively affect daily activities and life expectancy [2]. Current treatment strategies for CLTI, including stem cell therapies [3], mononuclear cell therapies [4], and growth factor-based treatments [5], have shown promise but failed to provide long-term solutions [6].

Bone marrow-derived mesenchymal stromal cells (BM-MSCs) have emerged as a potential therapeutic option for ischaemic diseases due to their regenerative properties [7], particularly their ability to secrete bioactive molecules that promote tissue repair. These secreted factors are thought to contribute to the paracrine effects of MSCs in promoting angiogenesis [8, 9]. Among these factors, extracellular vesicles (EVs) have gained attention for their ability to encapsulate and transfer bioactive molecules, including proteins and nucleic acids, to neighbouring cells [10, 11].

EVs derived from MSCs contain miRNAs that induce angiogenesis, known as angio-miRNAs [12], which regulate gene expression post-transcriptionally and promote endothelial cell growth, survival, and migration [13]. Among these, miRNA-126, -135b, and -210 are promising candidates for future clinical applications [14, 15].

In this study, we aimed to investigate whether EVs derived from genetically modified BM-MSCs overexpressing specific angio-miRNAs could enhance angiogenesis both *in vitro* and *in vivo*. Our goal was to develop angiogenic oligonucleotide therapeutics, progressing toward first-in-human studies and contributing to preventing limb amputation in patients with CLTI. Moreover, we sought to evaluate whether EV-based treatment could offer a more sustainable and long-term solution for ischaemic conditions compared to conventional therapies.

## Methods and materials

### Culture of BM-MSCs

The use of human BM-MSCs (lot no. TL281098; Lonza, Basel, Switzerland) was authorised by the provider. Experiments using human cells were approved by The Tokyo Medical University Medical Ethics Committee and the Regulations Concerning Medical Research (D23-036). Briefly, cells were seeded at a density of 2.0 × 10^5^ per 10-cm dish in 8 mL of MesenPro (Thermo Fisher Scientific, MA, USA) medium supplemented with 1% GlutaMAX™ (Thermo Fisher Scientific) and incubated at 37 °C and 5% CO_2_. BM-MSCs from the fourth passage were used for the experiments.

### Generation of BM-MSCs overexpressing angio-miRNAs

A lentiviral system was used to overexpress miRNA-126, -135b, and -210 in MSCs. Briefly, lentiviral stocks containing human miRNA precursors were obtained from BioSettia Inc. (CA, USA). MSCs were plated on 6-well plates at a density of 1.0 × 10^5^ cells/well and incubated for 24 h. Subsequently, the spent medium was replaced with a medium containing TransDux™ (System Biosciences, CA, USA), and MSCs were incubated with the lentiviral particles for 24 h. MSCs in the lentiviral vector group were transfected with lentiviral vectors containing miRNA-126, -135b, and -210 at a multiplicity of infection (MOI) of 1. MSCs in the control vector group were infected with an empty control vector at the same MOI. After transfection, the cell pools were selected using a medium containing 2.0 μg/mL puromycin for 3 days. The resulting genetically modified cells were named MSC-miRNA126, MSC-miRNA135b, MSC-miRNA210, and MSC-miRNA control.

### miRNA extraction and quantitative polymerase chain reaction (qPCR)

Total miRNAs of cultured MSCs were extracted using an miRNeasy Kit for miRNA Purification (Qiagen, Venlo, Netherlands) following the manufacturer’s protocol. cDNA synthesis was performed using a TaqMan™ MicroRNA Reverse Transcription Kit (Thermo Fisher Scientific). Real-time qPCR was performed using primers specific for miRNA-126, -135b, and -210. *U6* was used as an internal control.

### Separation and characterisation of EVs

The separation of EVs was conducted as follows. Briefly, 7 × 10^5^ BM-MSCs were passaged in a 15-cm dish containing 20 mL of StemPro (Thermo Fisher Scientific). After 24 h, the spent BM-MSC culture medium was collected and centrifuged at 2000×*g* for 10 min. The supernatant was then filtered through a 0.22-μm pore membrane filter and poured into 13.2-mL Open-Top Thinwall Ultra-Clear Tubes (Beckman Coulter, CA, USA). The tubes were ultracentrifuged using an SW 41 Ti rotor (Beckman Coulter) at 210,000×*g* and 4 °C for 70 min. The supernatant was decanted, and the precipitated EVs were diluted with phosphate-buffered saline (PBS(–)). The EV solution was then ultracentrifuged under the same conditions, the supernatant was decanted, and the pellets were collected. Each type of EV isolated was named after the vector transfected into its BM-MSC source (EVcontrol, -126, -135b, and -210); EVs derived from unmodified BM-MSCs were named EVNative. The concentration of EV-associated proteins was measured using a Qubit Fluorometer (Thermo Fisher Scientific). The particle number and size distribution of EVs were analysed by dynamic light scattering using a NanoSight instrument (Spectris, London, UK) following the manufacturer’s instructions. The ExoScreen assay was performed as previously described to capture CD63 [16]. These EV analyses were performed following the minimal information available for studies on EVs (MISEV2023) [17].

### Tube formation assay

Human umbilical vein endothelial cells (HUVECs; Lonza) were seeded at a density of 2.0 × 10^5^ cells per 10-cm dish in 8 mL of EBM-2 medium (Lonza) and incubated at 37 °C and 5% CO_2_. Cells from passages five to nine were used for the tube formation assay. HUVECs and EVs from BM-MSCs (0.5, 1, or 10 μg/mL) suspended in EBM-2 medium were seeded on top of a Matrigel layer (Corning, NY, USA) in each well of a 24-well plate at a density of 1.5 × 10^4^ cells and incubated at 37 °C for 24 h. In the control group, HUVECs were seeded onto Matrigel in PBS(–). Images were captured using a BZ-X 800 phase contrast microscope (Keyence, Osaka, Japan), and tube formation was analysed using the Angiogenesis Analyzer in ImageJ software (version 1.54d; https://imagej.net/; National Institutes of Health, Bethesda, MD, USA). The number of junctions, total length of segments, and total mesh areas counted by the software were compared.

We also performed tube formation analysis of HUVECs cultured with single or combined EVs (1 μg/mL) derived from transfected BM-MSCs. Combined EVs refer to a mixture of two or three EVs (EV126 and EV135b; EV135b and EV210; EV126 and EV210; and a combination of EV126, EV135b, and EV210). To analyse the angiogenic capacity of the BM-MSC culture medium, a non-contact co-culture model was established by placing a Transwell insert (Corning) in each well. Briefly, 1.0 × 10^4^ BM-MSCs were seeded in Transwell plates containing MesenPro medium. Tube formation by HUVECs was analysed after they were cultured separately from BM-MSCs in Transwell plates for 24 h. The number of junctions, total length of segments, and total mesh areas were compared between the native EVs, transfected EVs, and co-culture models.

### Mouse hindlimb ischaemia model

All animal experiments were approved by the Institutional Animal Care and Use Committee of Tokyo Medical University (R6-020). All animal studies were conducted in accordance with the National Institutes of Health Guide for the Care and Use of Laboratory Animals. This work followed the ARRIVE guidelines 2.0. After the experimental procedures, the mice were euthanised via inhalation of pure CO_2_ gas in a euthanasia chamber.

Briefly, 10-week-old male BALB/c mice were purchased from Japan SLC, Inc. (Hamamatsu, Japan). The mice were allocated to 11 groups with 4 mice per group: sham surgery, PBS(–), native EVs, control EVs, single EVs (EV126, EV135b, or EV210), double EVs (EV126 and EV135b, EV135b and EV210, or EV126 and EV210), and triple EVs (EV126, EV135b, and EV210). The mice were then anaesthetised via intraperitoneal injection of a mixture of midazolam (0.4 mg/mL), butorphanol (0.5 mg/mL), and medetomidine (0.075 mg/mL). Both hindlimbs were fixed in the supine position with extension and abduction. An oblique incision was made on the left groin, and the subcutaneous fat pad was then dissected. The femoral artery, vein, and nerve were identified and separated gently. The common femoral artery was ligated at two points using 6–0 polyvinylidene fluoride sutures (Kono Seisakusho, Ichikawa, Japan).

Unilateral hindlimb ischaemia was induced by excising the left common femoral artery between the two ligations. After the excision of the common femoral artery, 50 μL of EVs containing 4 μg protein were injected into the extensor and flexor muscles of the thigh from the surgical site. PBS(−) was injected into the mice in the control group. The incision was closed using 4–0 silk sutures (Kono Seisakusho). The right hindlimb was used as the internal control. Mice were placed on a heating pad maintained at 37 °C during the procedure. Four mice underwent a sham operation in which the left common femoral artery was exposed but not ligated.

### Evaluation of limbs

The severity of the ischaemic injury was evaluated based on the grade of limb necrosis according to a previous study [18]: grade 0, normal limb without necrosis; grade I, black toenails with necrosis limited to the toes; grade II, necrosis extending to the foot; grade III, necrosis extending to the knee; and grade IV, necrosis extending to the hip or loss of the entire limb.

Blood flow was monitored using a MoorFLPI-2 instrument (Moor Instruments, Devon, UK) on days 0 (before and after the procedure) and 7. To evaluate the alterations in limb perfusion, the blood flow ratio of the left to the right foot was analysed. The region of interest was set at the ankle, which reflected peripheral blood perfusion.

### Histological and immunohistochemical analyses

Limb muscle tissues were immunohistochemically stained with haematoxylin–eosin and anti-CD31 antibodies (Cat. no. GTX130274, GeneTex International Corporation, CA, USA). Briefly, the left hamstrings were harvested from the mice in each group on day 14 (n = 1 per group) and fixed with a 10% formalin solution (Muto Pure Chemicals, Tokyo, Japan). The samples were then embedded in paraffin, and 3-μm-thick serial sections were processed for haematoxylin–eosin staining.

For immunostaining to detect CD31, sections were incubated with anti-CD31 antibodies (1:4000) overnight at 4 °C and subsequently incubated with the secondary antibody Histofine Simple Stain Mouse MAX-PO^®^ (1:1; Nichirei, Tokyo, Japan) for 30 min at 25 °C. To evaluate angiogenesis in ischaemic muscle, we compared the ratio of the area occupied by capillary endothelial cells in ischaemic muscle between mice injected with EVs and PBS(–). The areas of the muscle and capillary endothelial cells were calculated using ImageJ software.

### Statistical analyses

The results are expressed as the mean ± standard error of the mean. Differences between groups were determined using analysis of variance (ANOVA). All data were analysed using EZR (Saitama Medical Center, Jichi Medical University, Saitama, Japan), a graphical user interface for R (R Foundation for Statistical Computing, Vienna, Austria). Statistical significance was set at *p* < 0.05.

## Results

### Establishment of transfected MSCs

**Fig. 1:**
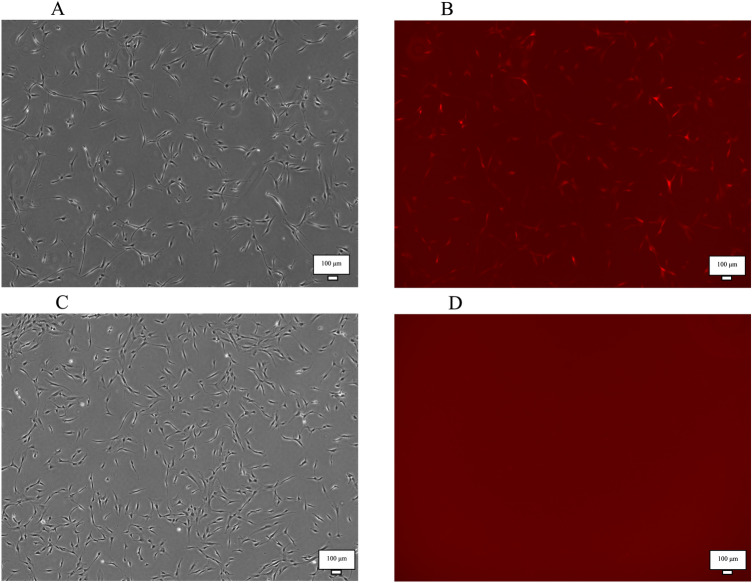
Transfection of bone marrow-derived mesenchymal stromal cells (BM-MSCs). **A** BM-MSCs transduced with lentiviral vectors. **B** Red fluorescent protein (RFP) fluorescence shows that the vector is integrated into the BM-MSC genome. **C, D** Native BM-MSCs do not express RFP

Successful miRNA transfection in MSCs was confirmed via red fluorescent protein (RFP) expression (Fig. 1). qPCR analyses showed a 395.2-fold change in the expression of miRNA-126 in MSC-miRNA126 relative to that of *U6*, whereas the expression of miRNA-135b in MSC-miRNA135b was 132.4, and that of miRNA-210 in MSC-miRNA210 was 8.84 (Table 1). The differences in overexpression levels might have reflected inherent variability in miRNA incorporation and transcriptional regulation. However, the fold changes in the MSC-miRNA control did not change compared to that of U6.

**Table 1:** Relative gene expression of angio-microRNA (miRNA) in modified mesenchymal stromal cells (MSCs) compared with native MSCs

Fold change	miRNA-126	miRNA-135b	miRNA-210
MSC-miRNA control	0.8584	1.041	1.082
MSC-miRNA126	395.2	0.8424	1.214
MSC-miRNA135b	0.7409	132.4	1.137
MSC-miRNA210	0.1828	0.443	8.842

Fold changes were calculated relative to U6 expression

### Characterisation of EVs

**Fig. 2:**
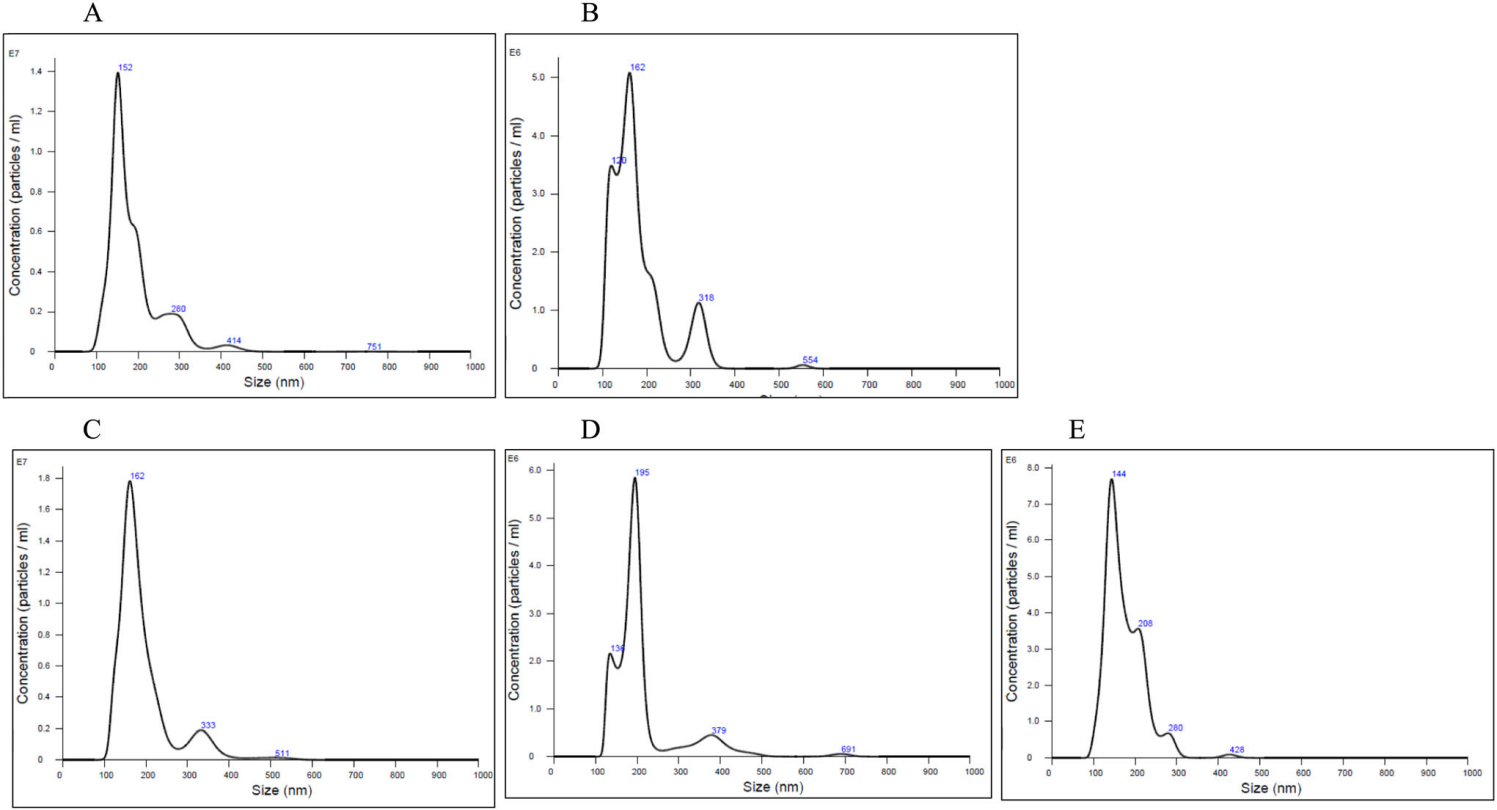
Particle size distribution in EV samples derived from each type of modified MSC. **A** EVNative. **B** EVcontrol. **C** EV126. **D** EV135b. **E** EV210. EV, extracellular vesicle; MSC, mesenchymal stromal cell. EV native refers to EVs from unmodified MSCs. EVcontrol refers to EVs from MSCs transfected with an empty control vector

**Fig. 3:**
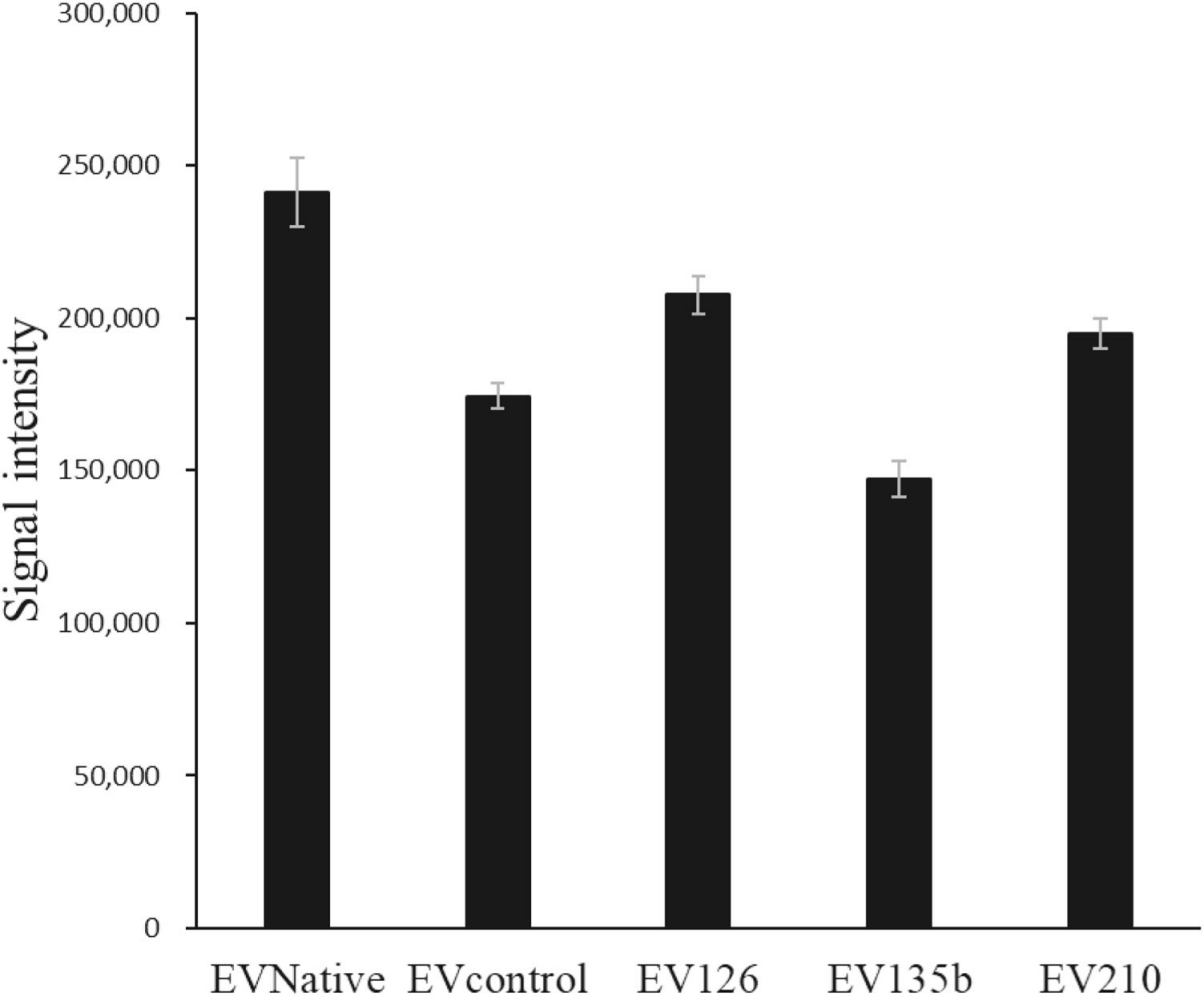
Results of the ExoScreen assay. Data are presented as the mean ± standard error of the mean (n = 3 samples for each group)

The volume of the culture medium and the total cell number used to isolate EV are presented in Table 2. Although the culturing time was consistent, the total cell number and medium volume varied among groups. This affected the yield of EVs in terms of both particle number and protein content. The particle concentrations and size distributions are presented in Table 3 and Fig. 2, respectively. The concentration of each EV was proportional to the total cell number. The protein concentrations of each EV and the protein-to-particle ratios are listed in Table 4. Specifically, the protein-to-particle ratios of EVNative, EVcontrol, EV126, EV135b, and EV210 were 1.71 × 10^−8^, 1.68 × 10^−8^, 8.74 × 10^−9^, 1.20 × 10^−8^, and 1.31 × 10^−8^, respectively. ExoScreen analysis confirmed the presence of CD63 in all the samples (Fig. 3). These findings indicate that the quality of EVs derived from BM-MSCs aligned with the MISEV2023 standards.

**Table 2:** Culture medium and total cell number used for extracellular vesicle separation

	Culture medium (mL)	Total cell number
EVNative	272.6	9.17 × 10^6^
EVcontrol	179.4	6.50 × 10^6^
EV126	220.8	9.22 × 10^6^
EV135b	218.2	0.88 × 10^6^
EV210	186.4	8.60 × 10^6^

**Table 3:** Particle size distribution and concentration measured using NanoSight

	Mean, (nm)	Mode, (nm)	SD, (nm)	D10, (nm)	D50, (nm)	D90, (nm)	Concentration, particles/mL
EVNative	189	151.6	63.4	132.5	168.7	282.9	1.02 × 10^11^
EVcontrol	179.7	161.3	65.3	117.7	162.9	299	4.41 × 10^10^
EV126	193.4	161.4	68.1	135.7	173.2	301.2	1.39 × 10^11^
EV135b	212.4	194.6	85.9	140.6	191.6	353	3.67 × 10^10^
EV210	174.1	143.5	46.8	126.4	163.1	230.4	6.0 × 10^10^

EV, extracellular vesicle

**Table 4:** Protein concentration and protein-to-particle ratio of each type of extracellular vesicle

	Protein concentration (μg/mL)	Protein/particles (μg/particle)
EVNative	1714	1.71 × 10^−8^
EVcontrol	742	1.68 × 10^−8^
EV126	1216	8.74 × 10^−9^
EV135b	440	1.20 × 10^−8^
EV210	784	1.31 × 10^−8^

### EVs induced tube formation in HUVECs

**Fig. 4:**
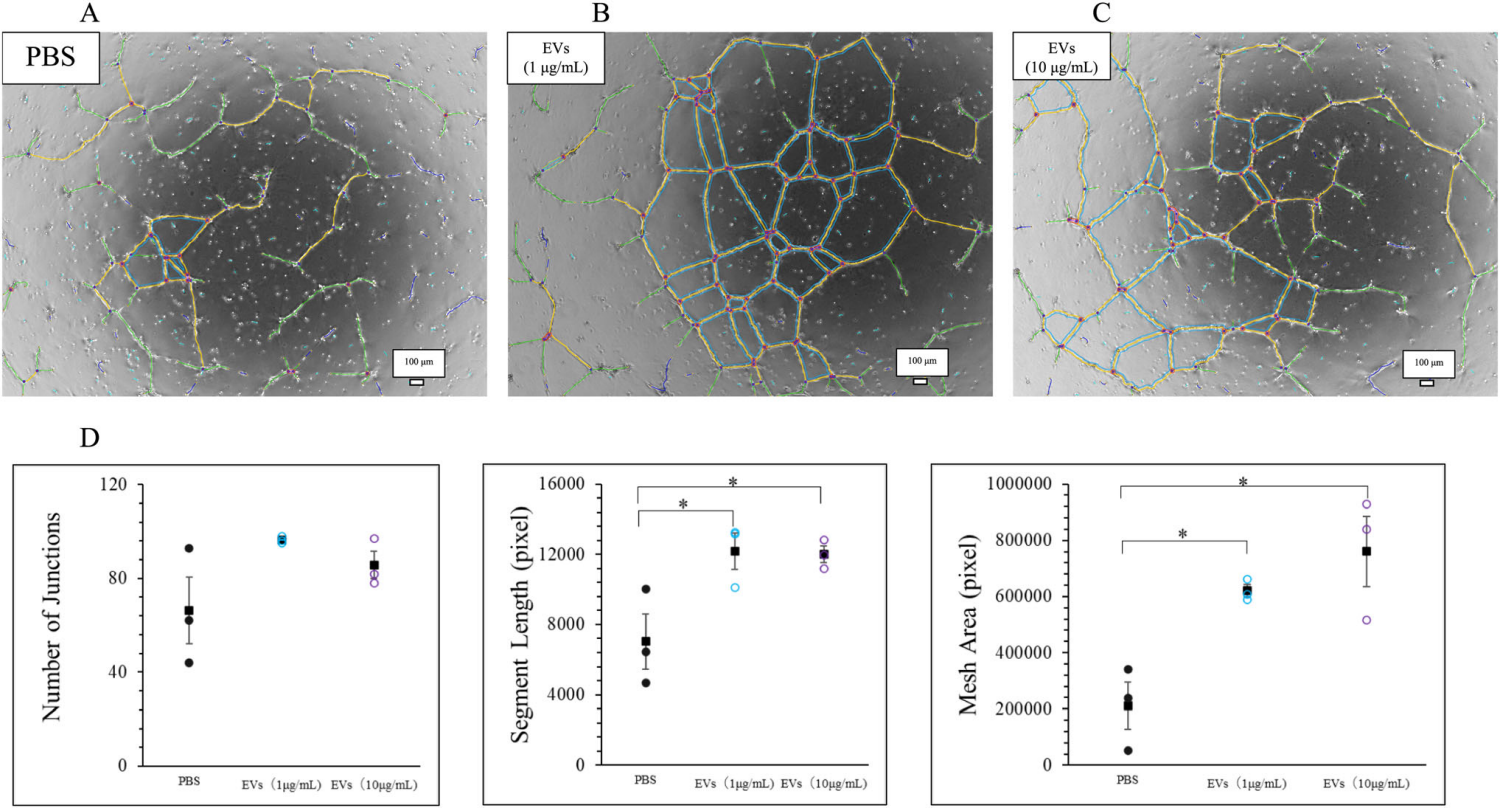
Analysis of tube formation in human umbilical vein endothelial cells (HUVECs) cultured with EVNative. **A–C** Representative images showing tube formation in HUVECs cultured with **A** phosphate-buffered saline, **B** EVNative, in which protein concentration was diluted to 1 μg/mL, and **C** HUVECs cultured with EVNative, in which protein concentration was diluted to 10 μg/mL. Red circles represent junctions. Yellow lines represent segments. Areas encircled by blue lines represent meshes. **D** Tube formation parameters compared between the groups (n = 3 samples for each group). Data are presented as the mean ± standard error of the mean. *p < 0.05 (one-way ANOVA). (Color figure online)

**Fig. 5:**
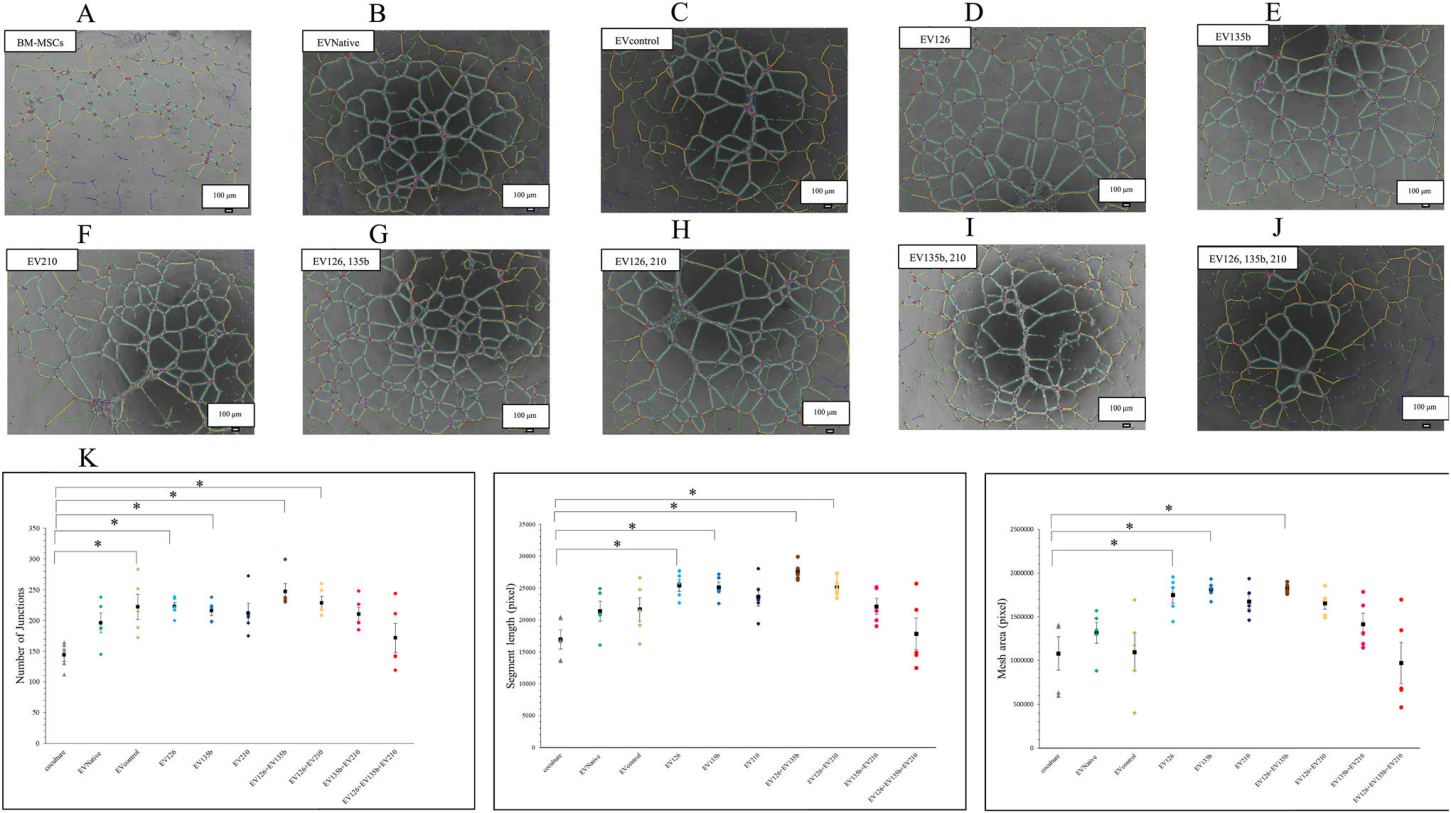
Analysis of tube formation in HUVECs co-cultured with BM-MSCs and EVs. **A–J** Representative images showing tube formation in HUVECs co-cultured with **A** BM-MSCs, **B** EVNative, **C** EVcontrol, **D** EV126, **E** EV135b, **F** EV210, **G** EV126 and EV135b, **H** EV126 and EV210, **I** EV135b and EV210, and **J** EV126, EV135b, and EV210. Red circles represent junctions. Yellow lines represent segments. Areas encircled by blue lines represent meshes. **K** Comparison of tube formation parameters between the groups (n = 5 samples in each group). Data are presented as the mean ± standard error of the mean. *p < 0.05 (one-way ANOVA). HUVECs, human umbilical vein endothelial cells; BM-MSCs, bone marrow-derived mesenchymal stromal cells; EVs, extracellular vesicles. (Color figure online)

Tube formation in HUVECs cultured with and without EVNative was analysed using ImageJ software (Fig. 4A–C). The findings showed a significant increase in tube formation parameters when HUVECs were cultured with EVNative (*p* < 0.05) (Fig. 4D). A comparison of the tube formation abilities of HUVECs cultured with single EVs, combined EVs, and BM-MSCs showed that EV126, EV135b, and EV126 + EV135b significantly enhanced the three tube formation parameters compared to co-culture with BM-MSCs alone (*p* < 0.05) (Fig. 5A–K). Moreover, EVs derived from transfected BM-MSCs increased tube formation compared with EVNative, although this was not significant (Fig. 5K).

### Hindlimb ischaemic model mouse establishment

**Fig. 6:**
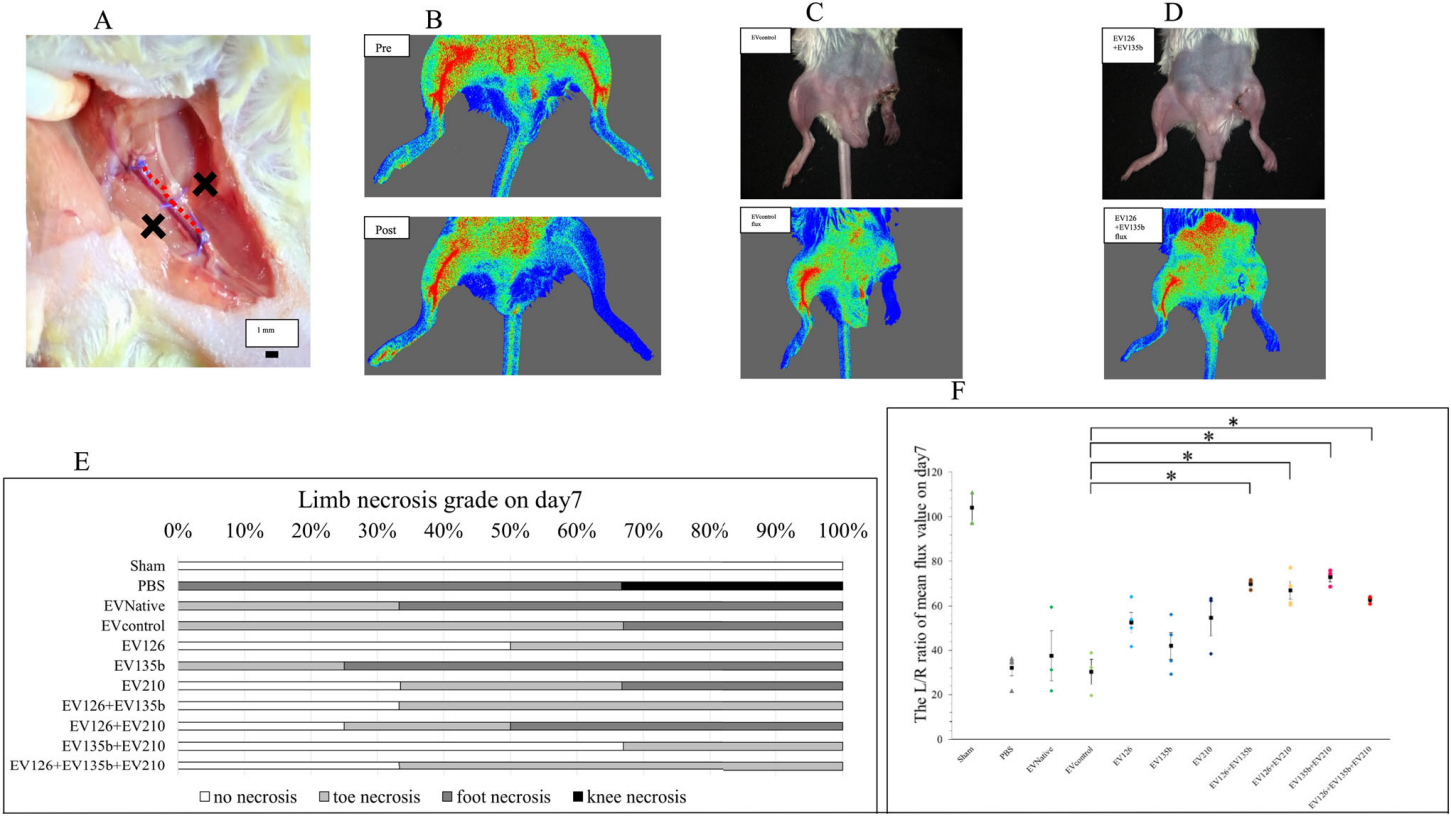
Mouse hindlimb ischaemia model. **A** Ligation and dissection of the left femoral artery. The red dotted line indicates the location of the femoral artery. Black crosses represent injection sites. **B** Measurement of limb perfusion pre- and post-procedure using Moor FLPI-2. **C** Hindlimb in an ischaemic mouse injected with the EVcontrol showed foot necrosis. **D** Hindlimb in an ischaemic mouse injected with combined EVs (EV126 and EV135b) did not show necrosis. **E** Grading of the severity of limb necrosis in each group. **F** Left-to-right ratio of the mean flux value on day 7. EVs, extracellular vesicles. (Color figure online)

After common femoral artery ligation and excision, 97.7% (43/44) of mice exhibited loss of toe perfusion (Fig. 6A, B). Limb necrosis grading on day 7 demonstrated that mice injected with the combined EVs had lower necrosis grades than mice injected with PBS (–), EVNative, or EVcontrol (Fig. 6C–E). Blood flow analysis on day 7 revealed no significant differences in limb perfusion between the EVNative and EVcontrol groups. Furthermore, limb perfusion in the double and triple EV groups recovered more significantly than that in the EVcontrol group (*p* < 0.05) (Fig. 6F).

**Fig. 7:**
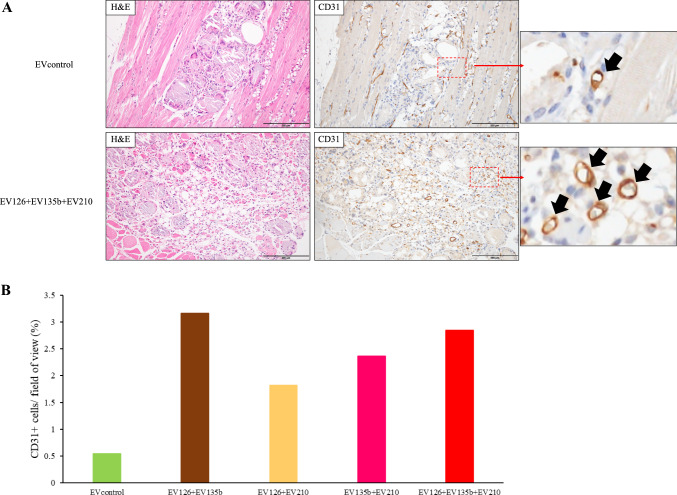
Histological analysis of the hamstrings of ischaemic hindlimbs (n = 1). **A** Representative image of haematoxylin and eosin and immunohistochemical staining for CD31. Arrows in higher magnified images marked newly formed capillaries. **B** Ratio of the area occupied by capillary endothelial cells in the ischaemic muscle

Immunohistochemical staining of muscle tissue revealed that the number of newly formed capillaries increased in the muscles injected with combined EVs compared to those injected with EVcontrol (Fig. 7A). Histological analysis showed that the ratio of capillary endothelial cells to the ischaemic muscle area was higher in mice injected with combined EVs than that in those injected with EVcontrol (Fig. 7B).

## Discussion

This study demonstrated that EVs enriched with specific angio-miRNAs enhanced angiogenic potency *in vitro* and *in vivo*. By leveraging genetically modified BM-MSCs, we successfully produced functionally enhanced EVs without compromising their fundamental properties. These findings provide new insights into EV-based cell-free angiogenic therapy to salvage ischaemic limbs from amputation.

One of the key challenges in EV-based research is heterogeneity, which can affect experimental reproducibility and data interpretation. To ensure standardisation, we adhered to MISEV2023 guidelines for isolation and use. Fewer cells were available for EV isolation in the MSC-miRNA135b group than those in the other groups due to slower proliferation, resulting in reduced protein and particle concentrations in the MSC-miRNA135b group; however, the particle size distribution and the protein-to-particle ratio were comparable across all groups. This finding suggests that although the total number of EV135b particles was reduced, the characteristics of the individual particles remained unchanged.

EVs derived from genetically modified BM-MSCs demonstrated superior angiogenic potency compared with those derived from BM-MSC co-cultures *in vitro*. Fukuda et al. conducted a clinical trial in which autologous BM-MSCs were injected into the critically ischaemic limbs, demonstrating the safety of BM-MSC-based therapy and improved limb amputation-free survival rates in patients [19]. However, while BM-MSCs exhibited angiogenic potential, only a small fraction differentiated into endothelial cells [20]. Instead, their angiogenic effects are primarily mediated by paracrine signaling [21]. Herein, we used a co-culture of BM-MSCs and HUVECs to simulate BM-MSC injection in a clinical trial. Our findings suggest that EVs derived from genetically modified BM-MSCs may serve as a potent alternative to BM-MSCs for therapeutic angiogenesis in patients with CLTI.

We also demonstrated that, when combined, EVs derived from genetically modified BM-MSCs exhibited higher angiogenic potency than the EVcontrol *in vivo*. MSC-derived EVs inherently possess angiogenic properties and hold promise for regenerative medicine applications [22]. Several strategies, including preconditioning, genetic modification of cells, and bioconjugation of EVs, have been explored to enhance the angiogenic potency of EVs [23]. Previous studies have shown that lentiviral vector transfection can induce the overexpression of specific miRNAs in BM-MSCs and that EVs harvested from these cells exhibit enhanced angiogenic effects [15]. Based on these findings, we hypothesised that combining multiple EVs containing different angio-miRNAs could enhance angiogenic potency to a greater extent than individual EVs. Our preliminary research indicated that BM-MSCs expressed miRNA-126, -135b, and -210 of major angio-miRNAs (data not shown). We generated three genetically modified BM-MSCs overexpressing one of these miRNAs and tested whether combinations of their derived EVs could promote angiogenesis. While we successfully demonstrated that combined EVs enhanced angiogenesis, the observed effects were not additive. This may be due to the fact that combinations of EVs inherently contain higher total amounts of miRNAs or particles than single EV preparations, thus complicating the interpretation of synergistic effects. An ideal alternative would be to engineer single MSCs to co-express two or three miRNAs, allowing EVs with uniform content to be isolated and dosed equivalently. In addition, interactions between angio-miRNAs have made the angiogenic potency more complicated. Although all three angio-miRNAs promote angiogenesis, they act via distinct pathways. MiRNA-126 regulates endothelial cell responses to vascular endothelial growth factor by repressing phosphoinositide-3 kinase regulatory subunit 2 and sprouty-related EVH1 domain-containing protein 1 [24]. MiRNA-135b promotes angiogenesis by inhibiting factor-inhibiting hypoxia-inducible factor-1 [25], whereas miRNA-210 facilitates angiogenesis by inhibiting Ephrin A3 [26]. The complex interactions between these angiogenic factors may explain why combined EV injections did not exert purely additive effects.

In this study, EVs were injected into the thigh muscles at the surgical site. The injection site and timing have not been strictly standardised in previous studies [27–30]. We selected this approach to allow for direct observation and precise injection. The high incidence rate of toe ischaemia following femoral artery excision, as confirmed by our MoorFLPI-2 results, validated the accuracy of our hindlimb ischaemia model. We also attempted to measure percutaneous oxygen saturation; however, the pulse wave was too weak to obtain reliable readings. Laser speckle imaging has been used to assess visceral perfusion [31, 32]. In our study, visual analysis of peripheral blood perfusion using MoorFLPI-2 provided a precise evaluation of reperfusion following EV injection.

This study has several limitations. First, the evaluation period for the ischaemic limb was relatively short. Each group initially included four mice, and muscle tissue was harvested on day 14 from one individual per group. We intended to extend limb perfusion evaluation to days 21 and 28; however, this was not feasible as the mice succumbed to limb gangrene. Second, we did not investigate protein or gene expression changes during the angiogenic process. Future studies should explore the molecular mechanisms underlying EV-mediated angiogenesis. Additionally, variability in EV isolation and the potential off-target effects of miRNA overexpression remain concerns. To improve the therapeutic potential of EVs, future research should focus on optimising delivery methods, evaluating long-term outcomes, and exploring alternative miRNA combinations.

In conclusion, this study demonstrated that EVs derived from genetically modified BM-MSCs, enriched with specific angio-miRNAs, significantly enhanced angiogenesis *in vitro* and *in vivo*. Our findings provide a foundation for developing advanced, cell-free regenerative strategies that could improve outcomes for patients with CLTI.

## Data Availability

The data that supports the findings in this study are available from the corresponding authors upon reasonable request.
